# Incidence of Pediatric Multiple Sclerosis in Iran within 2000-2019

**DOI:** 10.22037/ijcn.v16i1.35572

**Published:** 2022-01-01

**Authors:** Mohammad Mahdi NASEHI, Ali NIKKHAH, Mahmood MOOSAZADEH, Sasan SAKET, Reza ALIZADEH NAVAEI

**Affiliations:** 1 Pediatric Neurology Research Center, Research Institute for Children’s Health, Shahid Beheshti University of Medical Sciences, Tehran, Iran.; 2 Pediatric Neurology Department, Mofid Children’s Hospital, Faculty of Medicine, ShahidBeheshti University of Medical Sciences, Tehran, Iran; 3 Epidemiology and Gastrointestinal Cancer Research Center, Non-communicable Diseases Institute, Mazandaran University of Medical Sciences, Sari, Iran.; 4 Assistant Professor of Pediatric Neurology, Department of Pediatric Neurology, School of Medicine, Pediatric Neurology Research Center, Shohada-e-Tajrish Hospital, Mofid Children's Hospital, Shahid Beheshti University of Medical Sciences, Tehran, Iran.

**Keywords:** Incidence, Pediatric MS, Epidemiology, Iran

## Abstract

**Objectives:**

Due to a lack of data on pediatric multiple sclerosis (MS) epidemiology in Iran, this study aimed to determine the incidence rate of pediatric MS in Iran.

**Materials & Methods:**

All the data of the patients with MS registered in the Ministry of Health and Medical Education of Iran for 20 years were collected in this study; those born in 1982 and diagnosed with the disease and treated since 2000 were included in this study. Therefor The collected variables were patients’ age at the time of diagnosis, gender, year of diagnosis, urban or rural residency, and province of residence. Additionally, age-specific incidence rates per 100,000 of the population were calculated.

**Results:**

This study was performed on 4544 cases of pediatric MS within 2000-2019, of which 997 patients (21.9%) were male. The mean age of the patients with MS at the time of diagnosis was 14.3±4.6 years, and 4414 children (97.1%) lived in urban areas. The incidence rate of pediatric MS in Iran during 20 years increased from 0.26 per 100,000 of the population in 2000 to 1.53 in 2019.

**Conclusion:**

The incidence of pediatric MS in Iran is high, and the development of diagnostic practices in the past decade in Iran has contributed to the detection of this high incidence.

## Introduction

Multiple sclerosis (MS) is a chronic inflammatory disease of the central nervous system, in which there is demyelination and diffuse neurodegeneration in both gray and white matter of the brain, spinal cord, and optic nerves (1). Pediatric MS, is also referred to as pediatric-onset multiple sclerosis (POMS), early-onset MS, or juvenile MS, is commonly characterized as MS, beginning before 16 years of age, although sometimes this cutoff is before 18 years of age (2). The MS is a rare disease in children. A systematic review containing 19 population-based studies of MS within 1965-2018 identified 1439 individuals with MS aged ≤ 19 years (3). 

The overall incidence rate of POMS varied from 0.05 to 2.85 per 100,000 of the individuals aged ≤ 19 years and was less than 1 per 100,000 in most of the studies. The overall prevalence of this disease varied from 0.7 to 26.9 per 100,000 children. The differences in case ascertainment and ethnic and regional variations can indicate the broad range of incidence and prevalence rates. Other studies suggest that POMS accounts for approximately 5% of patients with MS (3, 4). The onset of MS in patients under 10 years of age is lower than 1% (5). The prevalence of pediatric MS in females is more than in males, and the ratio of females to males is 2.8:1 in children ≥ 12 years of age (3). 

Genetic susceptibility in combination with environmental triggers, including exposure to infectious agents and low serum vitamin D levels, had a main role in its pathogenesis (6). There are some differences between POMS and adult MS. Fatigue is one of the most common morbidities in children with MS (7-12). The prevalence of depression in children with MS ranges from 20% to 50% (7, 8, 10, 13-15) and is often associated with fatigue (9, 12). Cognitive impairments are common manifestations of MS in adults and are more prevalent in children with MS (16, 17). Pediatric MS can be diagnosed using the McDonald criteria (18, 19).

The lack of data on pediatric MS epidemiology in Iran encouraged the researchers to carry out the present study to evaluate the incidence rate of pediatric MS in Iran.

## Materials & Methods

This descriptive cross-sectional study was performed on all the data of the patients with MS registered in the Ministry of Health and Medical Education of Iran. The diagnosis of pediatric MS was considered based on age under 18 years at the time of diagnosis. Therefore, for a period of 20 years, those born in 1982 and diagnosed with the disease and treated since 2000 were included in this study. The diagnosis of MS was through clinical and magnetic resonance imaging assessments by clinicians. The collected variables were patients’ age at the time of diagnosis, gender, year of diagnosis, urban or rural residency, and province of residence. The collected data were analyzed using SPSS 23 software (version), and age-specific incidence rates per 100,000 of the population were calculated by the direct method.

## Results

**Figure 1:**
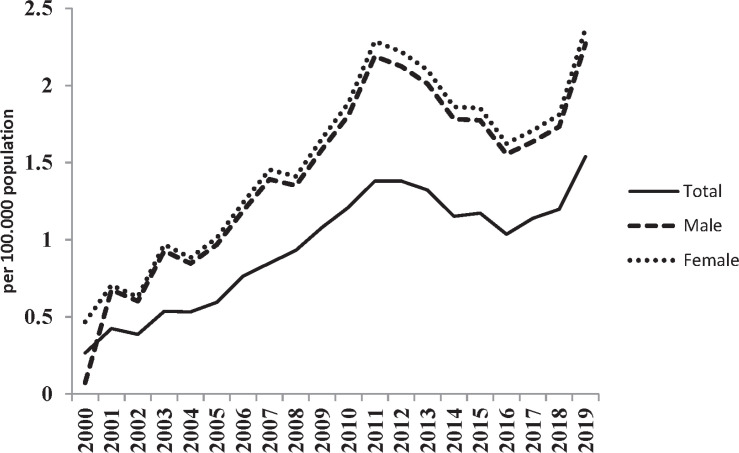
Incidence of Pediatric Multiple Sclerosis in Iran During 20 Years

**Figure 2:**
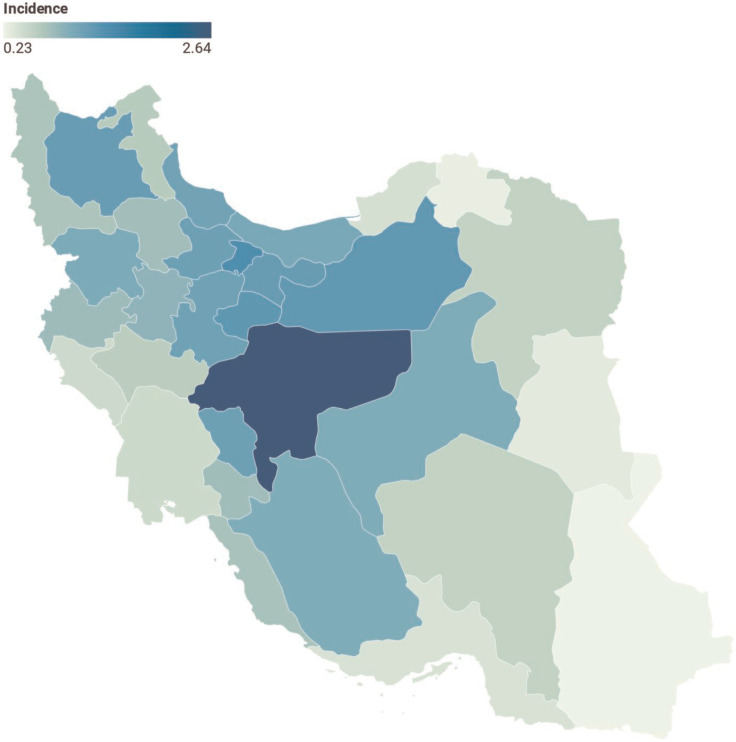
Distribution of Pediatric Multiple Sclerosis Incidence in Iran Provinces

This study was performed on 4544 cases of pediatric MS within 2000-2019, of which 997 (21.9%) and 3547 (78.1%) patients were male and female, respectively. Regarding residence, 4414 (97.1%) and 130 (2.9%) children lived in urban and rural areas, respectively. The mean age of the patients diagnosed with pediatric MS was 14.3±4.6 years (range: 1-18 years). Furthermore, the age of diagnosis was 5 years and less in 437 patients (9.5%), 6-10 years in 319 patients (7%), and 11 years in 3788 patients (83.4%). The number of new cases and incidence of pediatric MS in Iran increased from 64 in 2000 to 370 in 2019 (Table 1). The incidence rate of pediatric MS in Iran during 20 years increased from 0.26 per 100,000 of the population in 2000 to 1.53 in 2019. This rate increased from 0.46 to 2.37 and 0.7 to 0.74 per 100,000 individuals in females and males, respectively (Figure 1). The incidence rate of pediatric MS varied in different provinces of Iran and ranged from 0.23% in southeastern Iran (Sistan and Baluchestan province) to 2.63% in central Iran (Isfahan province) (Figure 2).

**Table 1:** Gender Distribution of Pediatric Multiple Sclerosis in Iran During 20 Years

Year	Female n (%)	Male n (%)	Total n (%)
2000	55 (85.94)	9 (14.06)	64 (100)
2001	83 (81.37)	19 (18.63)	102 (100)
2002	74 (79.57)	19 (20.43)	93 (100)
2003	114 (88.37)	15 (11.63)	129 (100)
2004	104 (81.25)	24 (18.75)	128 (100)
2005	119 (83.22)	24 (16.78)	143 (100)
2006	146 (79.35)	38 (20.65)	184 (100)
2007	171 (83.82)	33 (16.18)	204 (100)
2008	166 (74.11)	58 (25.89)	224 (100)
2009	195 (75.00)	65 (25.00)	260 (100)
2010	222 (76.29)	69 (23.71)	291 (100)
2011	269 (81.02)	63 (18.98)	332 (100)
2012	261 (78.61)	71 (21.39)	332 (100)
2013	247 (77.67)	71 (22.33)	318 (100)
2014	219 (79.06)	58 (20.94)	277 (100)
2015	218 (77.30)	64 (22.70)	282 (100)
2016	191 (76.71)	58 (23.29)	249 (100)
2017	201 (73.36)	73 (26.64)	274 (100)
2018	213 (73.96)	75 (26.04)	288 (100)
2019	279 (75.41)	91 (24.59)	370 (100)

## Discussion

This study aimed to investigate the incidence rate of pediatric MS in Iran, and the results showed that this rate was increased during 20 years and reached 1.53 per 100,000 of the population in 2019. This ratio is similar to that observed in a study conducted by Langer-Gould et al. (20) from the USA with an incidence rate of 1.66 per 100,000 of the population; however, it is lower than those in studies performed by Alroughani et al. (21) from Kuwait with a rate of 2.1 and Dell’Avvento et al. (22) from Italy with a rate of 2.58. Furthermore, the ratio calculated in the current study is higher than that in a study performed by Reinhardt et al. (23) from Germany, with an incidence rate of 0.64. Differences in diagnostic practices, population characteristics, and exposure to known risk factors might be responsible for these differences between the regions.

The results of the present study showed that in all the studied years, the incidence of pediatric MS in females was higher than in males (the male to female ratio: 0.28:1). Female predominance is a typical feature of adult MS (24). In this regard, the results of the current study are similar to the results of other epidemiological studies on pediatric MS (21, 23); nevertheless, in two studies carried out in the USA and Germany, the rate of pediatric MS in males was higher than in females (20, 22).

The mean age of pediatric MS diagnosis was 14.3±4.6 years, and the age of diagnosis was 5 years and under in 9.5% of cases and 11 years and over in 83.4% of the cases. Similar to the results of the present study, the mean age of pediatric MS diagnosis is within the range of 10-14 years in other studies (25, 26). However, in a large cohort from French and Belgian centers, the disease onset was at 10 years or younger in 7.6% of the cases (26), lower than that in the present study. 

The results of the present study showed that the incidence of pediatric MS varied in different provinces of Iran, with the lowest and highest ranges observed in Sistan and Baluchestan as a southeastern province and Isfahan as a central province, respectively. Moreover, these two locations had a 2.4% difference in the incidence of MS per 100,000 of the population. Iran is an extended country with different climates; accordingly, such a difference is expected, and environmental risk factors can be different between these provinces. Additionally, socioeconomic factors might be involved in MS risk (27), and the Human Development Index was different between Iranian provinces (28).

Concerning the limitations of the present study, it should be mentioned that this study was retrospective in nature, and diagnostic criteria were expected to be different in 2019 compared to those in 2000. Nevertheless, to the best of our knowledge, this study has been the first national study that addressed the demographic characteristics of pediatric MS patients, minimizing the selection bias observed in hospital- or clinic-based studies. It is required to perform further studies on the risk factors and other aspects of pediatric MS epidemiology. 

## In Conclusion

The incidence of pediatric MS in Iran is high, and the pediatric population needs to be given more attention and provided with better diagnostic and therapeutic services. Additionally, the rate of pediatric MS incidence was higher in females than in males and varied considerably between Iranian provinces.

## Author's contribution

Study concept, design, Interpretation, Drafting of the manuscript: Mohammad Mahdi Nasehi, Ali Nikkhah, Mahmood Moosazadeh, Sasan Saket, Reza Alizadeh-Navaei. Statistical analysis: Mahmood Moosazadeh and Reza Alizadeh-Navaei.

## Conflict of Interest

 None 
